# Native and Non‐Native Populations Respond Unevenly to River Barrier Removals

**DOI:** 10.1111/gcb.70941

**Published:** 2026-06-08

**Authors:** Ellen J. Dolan, Ismael Soto, Julian D. Olden, Jonathan D. Tonkin, Fengzhi He, Laís Carneiro, Jaimie T. A. Dick, Ross N. Cuthbert

**Affiliations:** ^1^ Institute for Global Food Security, School of Biological Sciences Queen's University Belfast Belfast UK; ^2^ University of South Bohemia in České Budějovice Faculty of Fisheries and Protection of Waters, South Bohemian Research Center of Aquaculture and Biodiversity of Hydrocenoses Vodňany Czech Republic; ^3^ Department of Conservation Biology and Global Change Doñana Biological Station(CSIC) Seville Spain; ^4^ School of Aquatic and Fishery Sciences University of Washington Seattle Washington USA; ^5^ School of Biological Sciences University of Canterbury Christchurch New Zealand; ^6^ Te Pūnaha Matatini Centre of Research Excellence University of Canterbury Christchurch New Zealand; ^7^ Key Laboratory of Marsh Wetland Ecosystem Conservation and Restoration, National Forestry and Grassland Administration Northeast Institute of Geography and Agroecology, Chinese Academy of Sciences Changchun China; ^8^ Université Paris‐Saclay, CNRS, AgroParisTech, Ecologie Société Evolution Gif‐sur‐Yvette France

**Keywords:** artificial barriers, biological invasion, dam removals, invasive species, meta‐analysis, populations, rivers, systematic review

## Abstract

The multi‐faceted threats of habitat degradation and biological invasions are increasing concerns for the integrity of freshwater ecosystems and their associated services globally. Removal of anthropogenic riverine barriers has gained momentum over the last three decades, motivated by the maintenance requirements of the aging infrastructure, while also functioning as a primary means of restoring river connectivity to promote ecosystem recovery and native biodiversity. Although barrier removals are intended to benefit native species dispersal, these actions can simultaneously facilitate non‐native species spread and increase biological invasion risk, creating a conservation paradox. Using a systematic review and meta‐analysis, we synthesised the patterns and trends in responses between native and non‐native populations to riverine barrier removals globally. Since 1998, research interest in the effects of barrier removals has rapidly accumulated, but with a major geographic imbalance towards North America (80% of studies). Fishes were the most studied taxon (74%), followed by riparian and aquatic plants (24%) and invertebrates (15%). The removal of dams dominated (85%), compared to road culverts (7%) and weirs (2%). Results from 2840 reported effect sizes showed that both native and non‐native population sizes generally increased after barrier removal, but the positive effect was strongest for native species. Moreover, these population responses were highly context‐dependent, with taxonomic grouping, sampling directionality and time since removal affecting outcomes. Non‐native population growth outstripped that of native species in the first few years post‐removal and non‐native invertebrates responded the most favourably to barrier removal. While native species typically benefit from barrier removals overall, our analyses highlight context‐specific responses that can simultaneously benefit non‐native populations and erode long‐term conservation outcomes. As river restoration efforts continue to gain traction globally, further planning and research are required to anticipate and assess long term population recoveries, to reconcile conservation quagmires around interacting global environmental changes.

## Graphical abstract

While barrier removals restore river connectivity and promote recovery of native populations, they may also facilitate non‐native species, creating a ‘connectivity conundrum’. Using a systematic review and meta‐analysis, we show that both native and non‐native populations can benefit from barrier removal, with outcomes being context‐dependent.

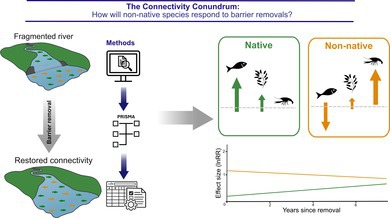

## 1 Introduction

Freshwater systems are home to at least 10% of all known animal species and more than 50% of all described fish species, despite covering less than 1% of the Earth's surface (Fricke et al. 2026; Strayer and Dudgeon 2010; Vardakas et al. 2025). Habitat fragmentation, in conjunction with other stressors, such as climate change, pollution and biological invasions, is a primary driver of global biodiversity loss (Brauer and Beheregaray 2020; Dudgeon 2019; Reid et al. 2019). Among freshwater ecosystems, rivers are especially susceptible to fragmentation due to their linear and dendritic nature (Sun et al. 2025; Vári et al. 2022). Artificial barriers in rivers are pervasive structures designed to manipulate flow, typically for anthropogenic water supply, flood control, hydropower generation or land use within the watershed. Across the world, these barriers affect longitudinal, lateral, vertical and temporal connectivity to varying degrees (Grill et al. 2019; Vári et al. 2022). Barriers, such as groynes, dykes and levees, are common fixtures which predominantly contribute to lateral (river channel‐floodplain) disconnection (Hermoso 2025; Thieme et al. 2024). Those which are most associated with longitudinal (i.e., headwater‐mouth) fragmentation include dams, weirs, culverts, fords (i.e., road crossings) and ramps (Boardman and Foster 2023; Couto and Olden 2018; Morden et al. 2022).

Today, more than 58,000 large dams and an estimated 4.4 million smaller structures fragment most of the world's rivers, with expectations that the number of barriers will increase globally (Couto and Olden 2018; Grill et al. 2019; Lehner et al. 2024; Zarfl et al. 2015). Numerous studies have shown that artificial barriers can degrade biotic and abiotic conditions by modifying downstream environments and impeding river connectivity in different dimensions (Borgwardt et al. 2019; Poff et al. 2007; He et al. 2024). The River Continuum Concept, proposed by Vannote et al. (1980) posits that geo‐ and hydro‐morphological characteristics of the watershed have predictable effects on community compositions and dynamics as well as ecological processes. This concept is foundational to the current understanding of freshwater ecology, underpinning much research and management (Doretto et al. 2020). An extension to the River Continuum Concept, entitled the Serial Discontinuity Concept, was developed by Ward and Stanford (1983) and models the extent and direction to which anthropogenic infrastructure disrupts river connectivity and the subsequent biotic characteristics. Since these concepts were developed, research interest in the effects of artificial barriers on biological communities has rapidly proliferated (Brodie et al. 2025; Firth et al. 2025).

Barriers affect biota by modifying habitat characteristics (e.g., suitability of flow and sediment conditions for spawning ground (Tang et al. 2022)), altering flow and thermal regimes (Poff et al. 2007; Olden and Naiman 2010), and reducing access to floodplain spawning and feeding grounds (Buddendorf et al. 2019; Fovet et al. 2023). Barriers, through effects on events such as flooding and droughts, cause alterations to the natural timings of key life history events impacting reproductive success and, in some cases, giving rise to behavioural adaptations (Bunn and Arthington 2002; Tonkin, Merritt, et al. 2018). Moreover, due to the heightened magnitude of human activities, barriers can act as hubs for the introduction of non‐native species, which are often generalists that exploit disturbed habitats below and above barriers (Dolan et al. 2025; Johnson et al. 2008).

Barriers can reduce longitudinal connectivity, leading to reduced range sizes, genetic isolation, artificial selective pressures on species traits, and altered behaviour (Dolan et al. in press; Jones, Champneys, et al. 2021; Zarri et al. 2022). The impacts of barriers via limited longitudinal connectivity on movements have been well described for many organisms, including plants (Jones et al. 2020), invertebrates (Panagiotou et al. 2022), and vertebrates (Barbarossa et al. 2020; Sonkar and Gaurav 2020). Nevertheless, concerns about barriers and mounting motivations for their removal have largely been sparked by the limitations they place on large, native, migratory fish with socio‐economic value (He et al. 2021; Huang and Li 2024).

**FIGURE 1:**
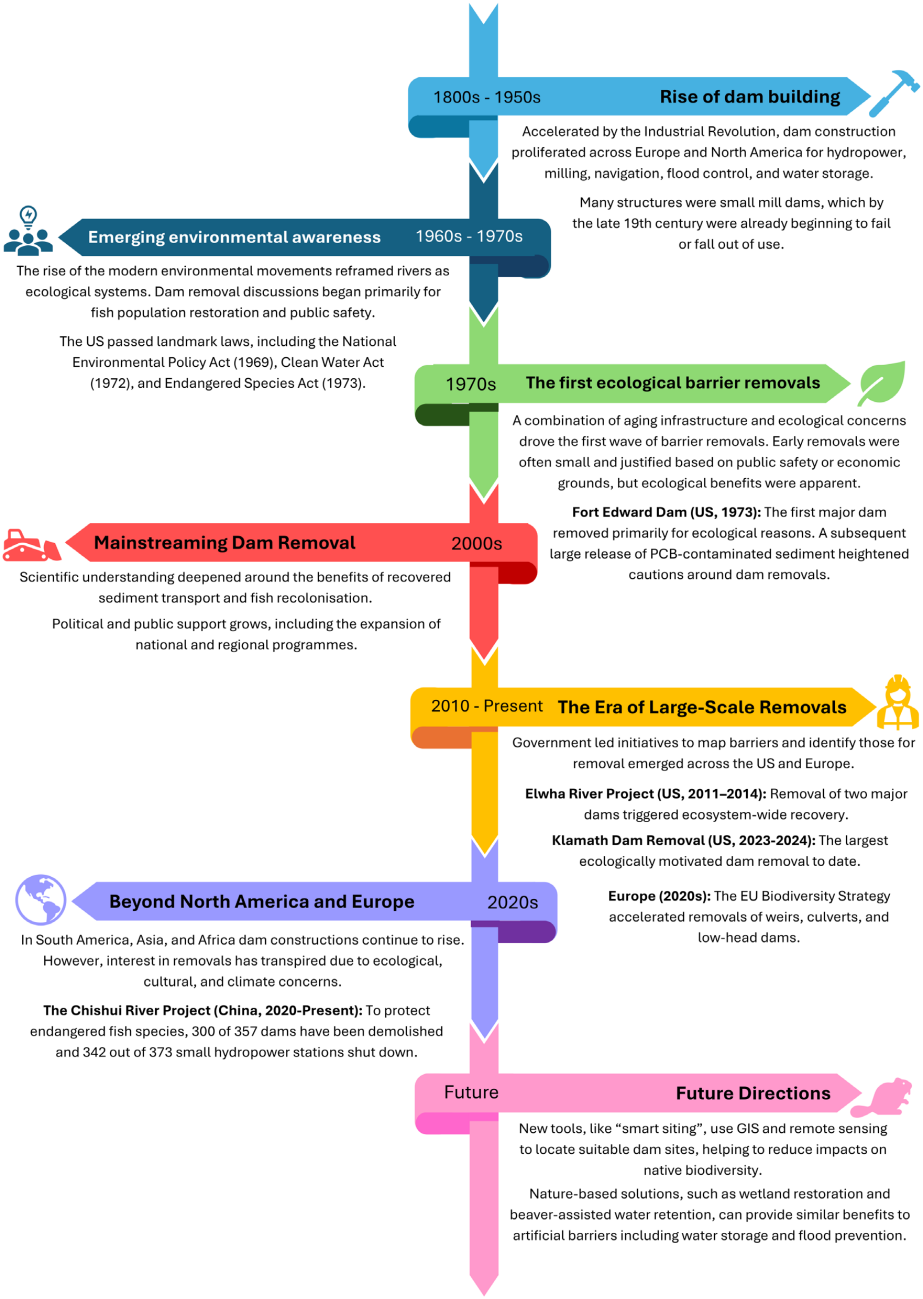
Conceptual timeline of the history of barrier removals globally.

Conservation strategies and policies focused on meeting freshwater restoration targets aim to tackle the issue of riverine fragmentation by removing longstanding barriers or enhancing movement via fish passage structures (Darre et al. 2025; McCaffery et al. 2024). The growing concern about the effects of barriers on habitats and the species they support gained momentum in the latter half of the 1900s (Figure 1) (Duda et al. 2023; Moore et al. 2021). Legislative movements have since filtered down to local councils and conservation groups, and have, for example, led to the removal of more than 2200 barriers within member countries of the European Union (EU) between 2020 and 2025 (Mouchlianitis 2026). Beyond ecological concern, many removal programmes have been motivated by the age and condition of the barrier and cost of repair (Dolan et al. 2025). In these cases, environmental restoration is often viewed as a beneficial byproduct, though this may lead to neglected monitoring and management of the biological community.

In tandem with growing concerns about the effects of riverine fragmentation, the presence and proliferation of non‐native species continues to rise across all taxonomic groups and regions, leading to a rapid expansion of research and public interest in the field of invasion science (Campbell and Simberloff 2022; Heger et al. 2024; Haubrock et al. 2026; Seebens et al. 2025). Biological invasions encompass the process of introduction, establishment and spread of non‐native species in a new region via anthropogenic pathways, with some of these species having impacts on the local ecosystem, human health or economy (Faulkner et al. 2024; IPBES 2023; Soto et al. 2024). Invasive species can have detrimental impacts on native species through mechanisms such as competition for resources, direct predation and disease transmission, with various degrees of impact depending on the level of ecological organisation (i.e., individual to ecosystem) (Carneiro et al. 2025; Ghia et al. 2025; Haubrock et al. 2024; Miller et al. 2024). Biological invasions can also cause substantial social, economic and cultural costs, reducing food security and recreational opportunities, altering culturally significant species and landscapes, modifying social interactions between local people and increasing risks to people's health and safety (Chen et al. 2026; Seebens et al. 2024). Moreover, conservation and management efforts can become complicated when invasive or naturalised species become integrated within local or Indigenous cultural identities, values or livelihoods (Jarić et al. 2025; Shackleton et al. 2019). The monetary burden of invasive species is estimated to cost global economies US$423 billion annually, with aquatic invasions accounting for a total of approximately US$345 billion between 1970 and 2020; although the true economic impact is likely much higher (IPBES 2023; Cuthbert et al. 2021; Soto et al. 2025).

Barriers have been widely regarded as an effective mechanism for slowing the spread of non‐native species (Dolan et al. 2025; Jones, Tummers, et al. 2021). Thus, reconnection of fragmented rivers may provide additional dispersal pathways for invasive species to take advantage of if they are not sufficiently planned for in removal strategies (Arkilanian et al. 2026). The passage of non‐native species may increase extinction risk and negate the positive potential of connectivity restoration (Bartholomew et al. 2023; Boardman and Foster 2023; Dolan et al. 2025; Rahel and McLaughlin 2018). For example, weirs have been shown to limit the upstream movement of the invasive Chinese mitten crab ( *Eriocheir sinensis* ) (Robinson et al. 2019), which is a highly aggressive invasive species that can drive top‐down trophic cascade effects through predation and disease transmission (Rosewarne et al. 2016; Schrimpf et al. 2014). Thus, longstanding barriers may intentionally or unintentionally limit the spread of economically and ecologically costly species, complicating barrier removal decision‐making.

While the removal of barriers provides an obvious solution to the restoration of riverscapes and movement corridors for spatially limited and isolated populations, it is emblematic of conservation challenges in the face of multiple stressors (Cooper et al. 2021; Dolan et al. 2025; Fraik et al. 2021). Managers must grapple with the need to remove barriers to enhance connectivity for native species while concurrently attempting to prevent the spread of invasive species that cause impact (Fausch et al. 2009). This isolation‐invasion dilemma—coined the ‘connectivity conundrum’ (Zielinski et al. 2020)—has been widely acknowledged by conservation biologists, yet in the growing landscape of barrier removal efforts, consistent integration of practical solutions to this risky trade‐off remains elusive (Bellmore et al. 2019). Where invasive species are removed or there are invasive species‐free habitats available beyond a barrier, coupling barrier removals and control of biological invasions can effectively restore native populations (Chenoweth et al. 2022; Marks et al. 2010). Many invasive freshwater species have been introduced intentionally, typically for coarse fishing or aquaculture, including salmonid and carp species (Bernery et al. 2024; MacNeil 2024). For reasons ranging from ethics to utilitarianism, diverging opinions have arisen regarding management and eradication efforts of these non‐native vertebrates, complicating conservation strategies (Banha et al. 2025). Exclusion and management of invasive species via barriers could also aid compromises between advocates for conserving and eradicating invasive species (Jones, Champneys, et al. 2021; Jones, Tummers, et al. 2021). Therefore, considering both phenomena can maximise conservation outcomes.

There remains a paucity of quantitative measures of population responses to barrier removals, hampering the development of comprehensive barrier removal planning and holistic risk assessment in the face of multiple stressors. Here, we synthesise and analyse the current knowledge around the magnitude, rate and direction of native and non‐native population changes associated with barrier removals. Our aims were (1) to systematically review publication trends regarding temporal data, geographic location, barrier typology, study design and biotic responses to barrier removals; and (2) to quantitatively measure these population level responses by comparing the effects on native and non‐native species.

## 2 Publication Patterns in Population Responses to Barrier Removals

### 2.1 Systematic Literature Review Methodology

We performed a systematic search across all Web of Science database collections (see search terms in Table S1) and selected papers published up to March 2025, following PRISMA (Preferred Reporting Items for Systematic Reviews and Meta‐Analyses) guidelines (Figure S1) (Moher et al. 2009; Page et al. 2021). We focused on primary research studies published in English; narrative reviews, meta‐analyses, prefaces and opinion articles were excluded. Searches specifically contained terms for longitudinal barriers. However, papers which considered the removal of barriers that primarily limit lateral flow were retained. All papers that conducted field surveys of biotic communities or populations of any species in the same river system as a barrier removal, whether before or after, were included for our systematic analysis (Table S2). After removing duplicates, a total of 1538 papers were retrieved and scanned by title and abstract using the pre‐defined inclusion and exclusion criteria (Table S2; Figure S1). Exclusion criteria were hierarchical; thus, papers were excluded based on the first reason they fulfilled. Three publications could not be obtained and thus were not included in our review (Figure S1). The 145 papers initially retained were subsequently screened and assessed based on our selection criteria, resulting in a final set of 45 papers (see Figure S1; Table S3). From these papers, we extracted a set of descriptor variables broadly encompassing barrier typology, taxonomic, invasional, temporal, geographic, methodological and study findings. These have been compiled into a novel database collating native and non‐native responses to barrier removals (see Table S4 for descriptors). Taxonomic classifications were extracted from the paper and standardised following the *Global Biodiversity Information Facility* (GBIF) backbone. Where taxonomy could be determined to species level, native and non‐native status (henceforth, ‘invasion status’) in the region of the study was assigned, whether provided in the paper or from the *Global Register of Introduced and Invasive Species* (GRIIS) and the *Global Invasive Species Database* (GISD). Where species level information was not available, taxa were assigned ‘unknown’ invasion status but were classified under their coarse taxonomic groupings for the purpose of the systematic review.

### 2.2 Spatiotemporal Publication Trends

**FIGURE 2:**
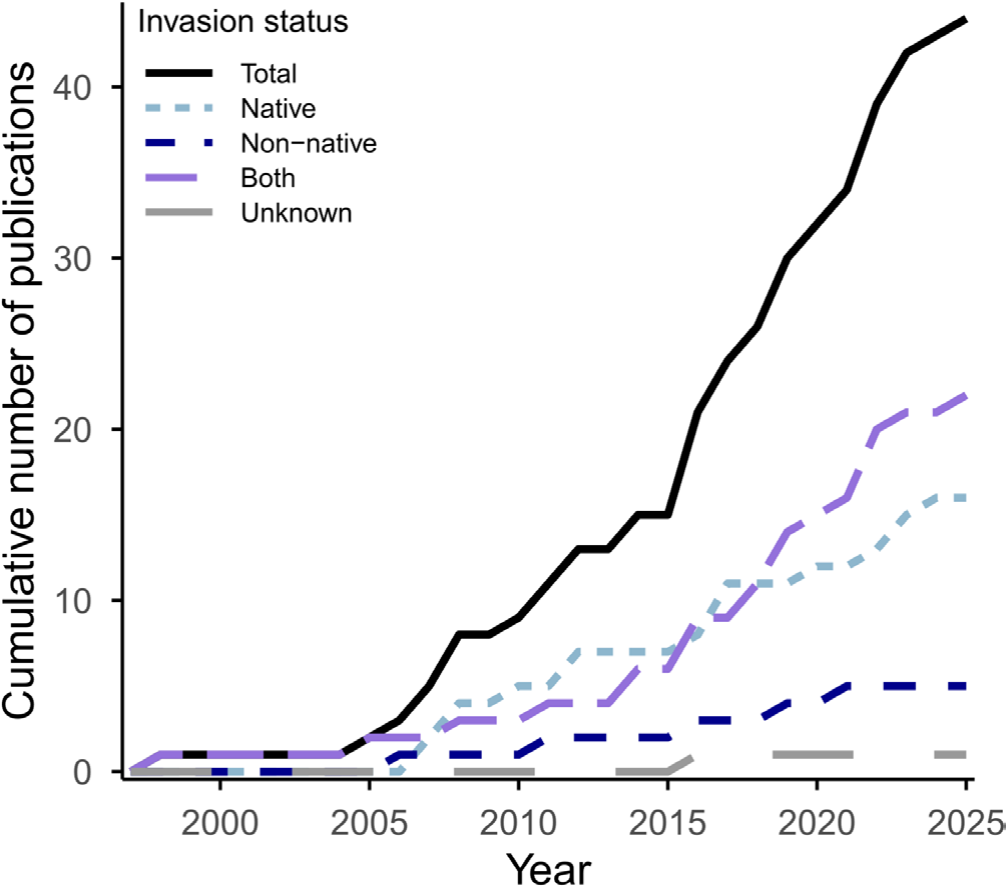
The cumulative number of publications which studied populations of native (light blue), non‐native (dark blue), both (purple) or species of unknown status (grey). The black line showed the cumulative total number of publications.

**FIGURE 3:**
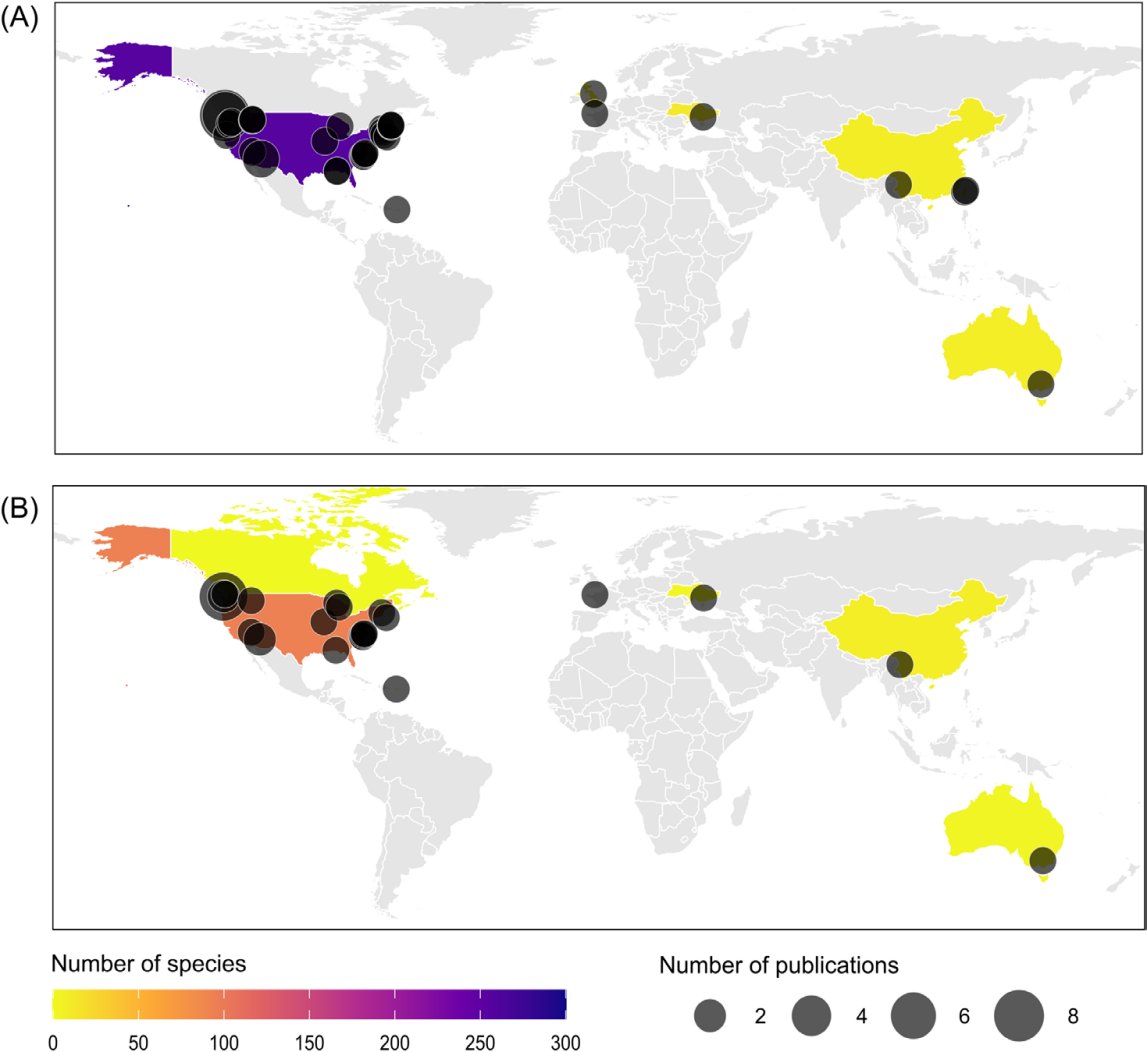
Map showing the number of (A) native and (B) non‐native species under study concerning barrier removals at the country level, with point size showing the number of publications around a barrier within a 10 km^2^ grid. Countries coloured in grey have no reported data.

The earliest publication in our dataset was from 1998, constituting the removal of a low‐head (i.e., less than 5 m tall) dam in a tributary of the Neuse River in North Carolina (United States (US)), studying both native and non‐native species. Since then, 40 publications included native species, and 28 included non‐native species, though these were mostly non‐independent (Figure 2). Publications were from 10 countries across four continents. The largest number of publications (*n*) came from North America (*n*_total_ = 37, *n*_native_ = 31, *n*_nonnative_ = 22), specifically the US (*n*_total_ = 35) (Figure 3). Four publications were from Europe (*n*_native_ = 4, *n*_nonnative_ = 3), three from Asia (*n*_native_ = 3, *n*_nonnative_ = 1) and one from Oceania (*n*_native_ = 1, *n*_nonnative_ = 1). Across the US, the number of different river systems studied remains limited (*n* = 21), with the largest share of publications hailing from the Elwha dam removal project (Nature 2018) in Washington State (*n* = 7) (Figure 3). Conspicuously, Africa and South America are absent from our database (Figure 3).

### 2.3 Barrier Removal Effects Across Taxa by Invasion History

**FIGURE 4:**
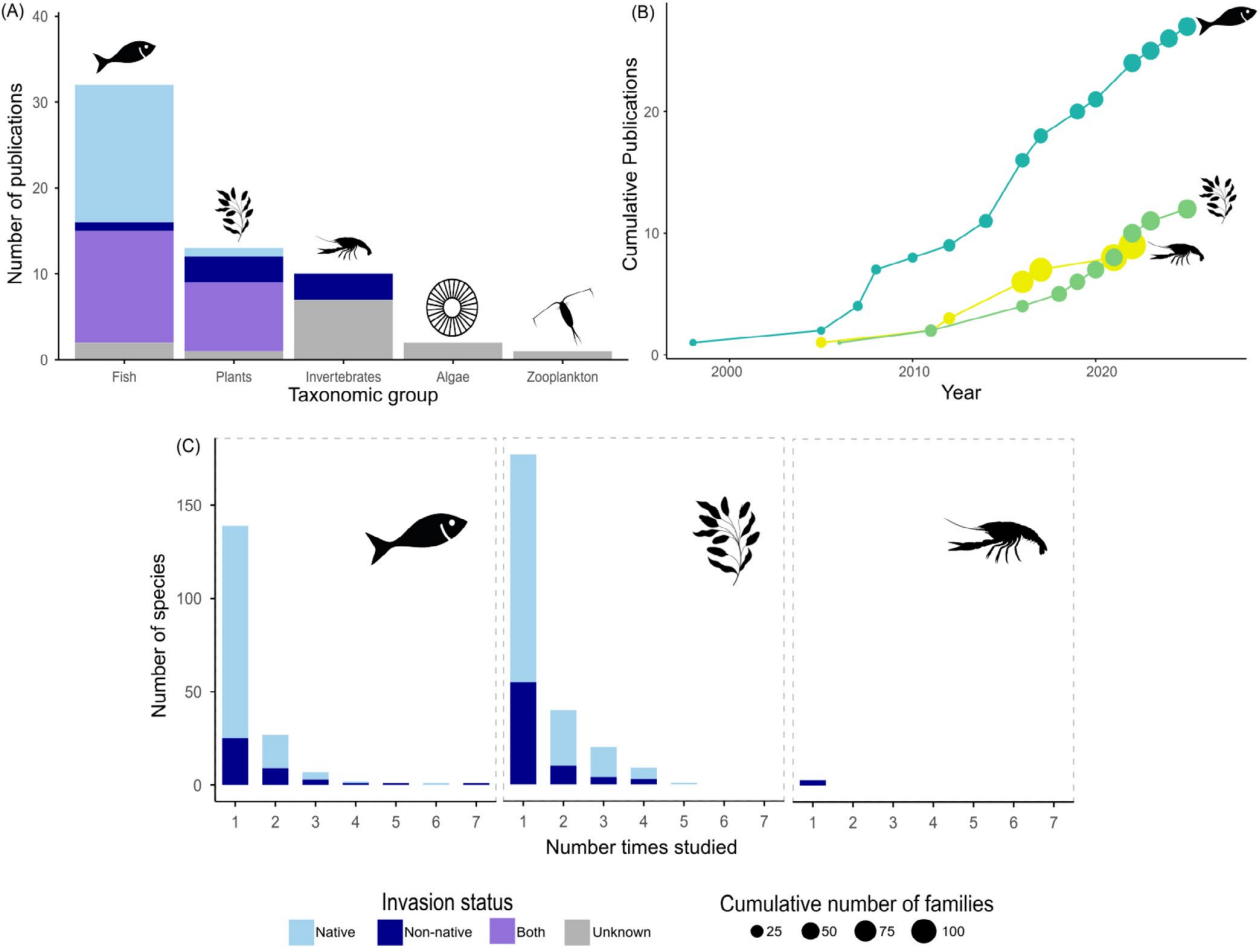
(A) The number of publications that study each taxonomic group, coloured by only native species (light blue), only non‐native species (dark blue), both native and non‐native species (purple) and unknown invasion status available (grey). (B) The cumulative number of unique families depicted by node size within each taxonomic group, along with the cumulative number of publications. (C) The number of publications in which each identified native (light blue) and non‐native (dark blue) species occurs, grouped by fish, plants and invertebrates (left to right). Notably, there were no native invertebrates identified to species level in any paper.

Taxonomic groups were unevenly studied across papers, including fish (*n* = 31; phylum: Chordata), invertebrates (*n* = 8; phyla: Acanthocephala, Annelida, Arthropoda, Cnidaria, Mollusca, Nematoda, Nematomorpha, Platyhelminthes) and plants (*n* = 13 papers; phyla: Anthophyta, Bryophyta, Pinophyta, Streptophyta, Spermatophyta, Tracheophyta), with single studies each on phytoplankton, zooplankton and benthic algae. The greatest family diversity (*D*) was among invertebrates (*D* = 80), followed by fish (*D* = 50) and plants (*D* = 46) (Figure 4). While fish were the most commonly studied taxonomic group, overall, the proportion of papers which focused on non‐native (*n*_nonnative_ = 1) relative to native (*n*_*native*_ = 16) fish was lower compared to those among plants (*n*_native_ = 4; *n*_nonnative_ = 3) and invertebrates (*n*_native_ = 0; *n*_nonnative_ = 3).

The two most studied fish species by invasion status across publications were non‐native common carp ( *Cyprinus carpio* ) (*n* = 7) and native rainbow trout ( *Oncorhynchus mykiss* ) (*n* = 6). The most studied plant was native red alder ( *Alnus rubra* ) (*n* = 5) and for non‐native plant species were Yorkshire fog ( *Holcus lanatus* ), wall lettuce ( *Mycelis muralis* ) and woodland ragwort ( *Senecio sylvaticus* ) (all *n* = 4). Only three invertebrates were identified to species level—virile crayfish ( *Faxonius virilis* ), pygmy peaclam (*Pisidium moitessierianum*) and New Zealand mudsnail ( *Potamopyrgus antipodarum* )*—*all of which were studied in their invasive range (all *n* = 1).

### 2.4 Understanding Approaches to Barrier Removals

Additional attributes of barrier removal include the dimension of the barrier and study design and duration; both important to contextualise their effects on the species and environment. The majority of publications consider dams (*n* = 39) rather than culverts (*n* = 3), levees (*n* = 2) and weirs (*n* = 1) in terms of their effects on riverine populations and communities. However, only half of studies (*n* = 22) reported the dimensions of barriers removed, with height being the most recorded (*n* = 20), followed by width (*n* = 9) and length of crest (*n* = 4). For seven of the publications that described dams, the available height measurement confirmed that they were low‐head—those less than five metres tall—structures.

**FIGURE 5:**
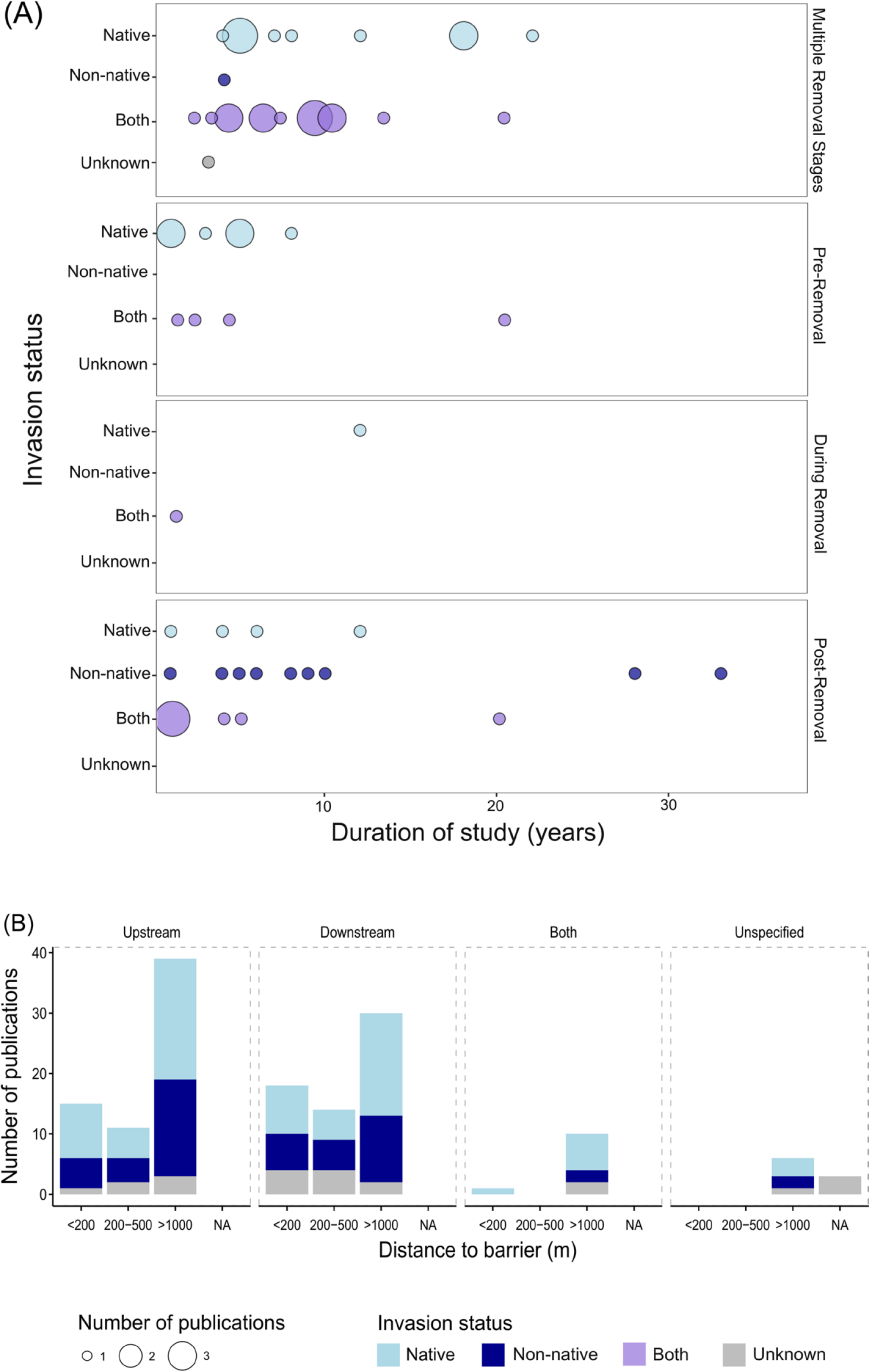
(A) The number of publications (dot size) across each stage of the removal process studied and the total duration of years the study was completed over. Any publications that studied at least two stages of removal (i.e., Pre–Post and/or Pre–Post) were grouped into ‘Multiple Removal Stages’. (B) The number of occurrences of native and non‐native populations in publications across each measured direction and distance relative to the barrier. Light blue represents native species and dark blue non‐native species. Publications were split into native and non‐native occurrences rather than grouped as ‘both’, as directionality and sampling distance may differ among sites within the same study.

The median study duration was 4 years, with most of the papers (*n* = 31) being undertaken for more than 1 year. Regarding experimental design, 24 publications included data from only a single stage of removal: pre (*n* = 12), the same year in which the barrier was removed (hereafter, during) (*n* = 2) or post (*n* = 10) removal data. Twenty‐one publications had data from multiple stages, including pre‐ and post‐removal (*n* = 16), pre‐ and during removal (*n* = 4), and during and post‐removal (*n* = 1) (Figure 5a). Interestingly, no publications studying only non‐native species were conducted solely pre‐ or during removal, with the majority of these papers only focused on post‐removal, signifying a reactionary rather than proactive response to biological invasions. We recorded the sample locations relative to the barrier, described in each publication, including upstream (*n* = 31), downstream (*n* = 25) and unknown direction (*n* = 8) (Figure 5b). Only 19 publications documented the distance from the barrier at which the samples were taken, of which 15 publications documented the distance upstream, 12 downstream and one in both directions.

## 3 Quantifying Native and Non‐Native Population Responses

### 3.1 Meta‐Analysis Methodology

Only those publications with a before‐after or before‐after‐control‐impact design that allowed for a reliable determination of the effect size were included in the meta‐analysis, that is, before versus after barrier removals (*n* = 21) (Table S3; Figure S1). Publications in which sampling was conducted in the same year as the barrier removal (i.e., referred to as ‘during’) but a different month were included in the meta‐analysis. Furthermore, only data where identification at the species level occurred were included, to determine the invasion status. To quantify the sign and strength of native and non‐native population responses, we calculated the effect size (Log Response Ratio; LRR) for each observation as the natural logarithm of the ratio between the most recent population size before the barrier removal and the population size for each year studied after the completion of the barrier removal. A small continuity correction (0.05) was added to zero counts (*P*_pre_ = 0 and *P*_post_ = 0) to allow for log‐transformation (Equation 1). Once corrected, the log response ratio was then calculated, where *y*_i_is the effect size (LRR) for observation *i*, P*_pre_ and P*_post_ are the continuity‐corrected pre‐ and post‐removal population values, respectively (Equation 2). The sampling variance *v_i_* of each LRR was approximated (Equation 3).

$$P_{\mathrm{pre}}^{*}=\left\{\begin{array}{c} P_{\mathrm{pre}}+c,\;\text{if}\;P_{\mathrm{pre}}\leq 0\\ P_{\mathrm{pre}},\;\text{if}\;P_{\mathrm{pre}}>0 \end{array}\right.$$

(1) $$P_{\text{post}}^{*}=\left\{\begin{array}{c} P_{\text{post}}+c,\;\text{if}\;P_{\text{post}}\leq 0\\ P_{\mathrm{pre}},\;\text{if}\;P_{\text{post}}>0 \end{array}\right.$$

(2) $$y_{i}=\ln\left(\frac{P_{\text{post}}^{*}}{P_{\mathrm{pre}}^{*}}\right)$$

(3) $$v_{i}=\frac{1}{P_{\text{post}}^{*}}+\frac{1}{P_{\mathrm{pre}}^{*}}$$

We conducted multilevel meta‐analyses using the restricted maximum‐likelihood (REML) estimator through the *rma.mv* function in the *metafor* package in R (v.4.5.1; R Core Team 2025) (Viechtbauer 2010). We assessed potential publication bias on our effect sizes using funnel plots to visually examine the distribution of effect sizes around the standard error, and Egger's linear regression to test for asymmetry by regressing the effect size estimates against the standard error for statistical testing (Egger et al. 1997; Nakagawa et al. 2022).

The model included invasion status [native (N) and non‐native (NN)] as a fixed moderator to test for differences in effect sizes between groups (see section 3.2). For subsequent models, the interactions between invasion status and years since removal (see section 3.3), taxonomic grouping (see section 3.4) and position of populations in rivers relative to the barrier removed (see section 3.5) were also included as fixed moderators (see details for all models in Table S5). We removed the intercept from models with categorical moderators to estimate the mean effect size for each combination of moderator levels directly, rather than expressing effects relative to a reference level. We separated the dataset into native and non‐native species effect sizes to analyse years since removal, allowing for distinct temporal trajectories to be determined without constraining the slopes for native and non‐native species to a common baseline, thereby facilitating direct interpretation of recovery rates within each invasion status category. For years since removal, we modelled the linear relationships between invasion status and years, as well as across binned year groups (0–1 year, 2–5 years and ≥ 6 years). The maximum number of years post‐removal studied was seven. Random effects were specified for observation ID nested within paper ID (to account for variations in approaches within and among studies) and sample site ID (to account for the non‐independence of multiple effect sizes reported within the same sampling site over years). Collinearity, which was measured using the *vif* function within the *metafor* package, was low among all models (all VIFs < 3.45) (Viechtbauer 2010). Model results were back‐transformed from the log response ratio to the response ratio for interpretation. Differences between the coefficients of native and non‐native species were tested using pairwise contrasts of a Wald‐type chi‐square test, via the *anova.rma* function in the *metafor* package.

### 3.2 Overall Population‐Level Responses to Barrier Removals

We fit an intercept‐only random‐effects model to estimate the overall effect size and quantified between‐study heterogeneity and variability (Table S5). Population size had a non‐significant tendency to increase after the removal of a barrier (*β* = 0.546, 95% CI = [−0.394, 1.46]) across all reported effects. There was high heterogeneity among effect sizes (*I* ^2^ = 93.1%), mostly from between‐studies variations (*I* ^2^ = 80.2%), compared to within‐study differences (*I* ^2^ = 11.0%) and repeated sample sites (*I* ^2^ = 1.9%). Asymmetry was observed in the funnel plots and Egger's tests were significant, indicating the presence of publication bias (*t* = 5.56, df = 2838, *p* < 0.001) (Egger et al. 1997; Nakagawa et al. 2022). However, the asymmetry detected in our funnel plots does not invalidate our analysis as they can arise from multiple sources other than publication bias, including chance and high heterogeneity among studies, as in our case (Sterne et al. 2011).

**FIGURE 6:**
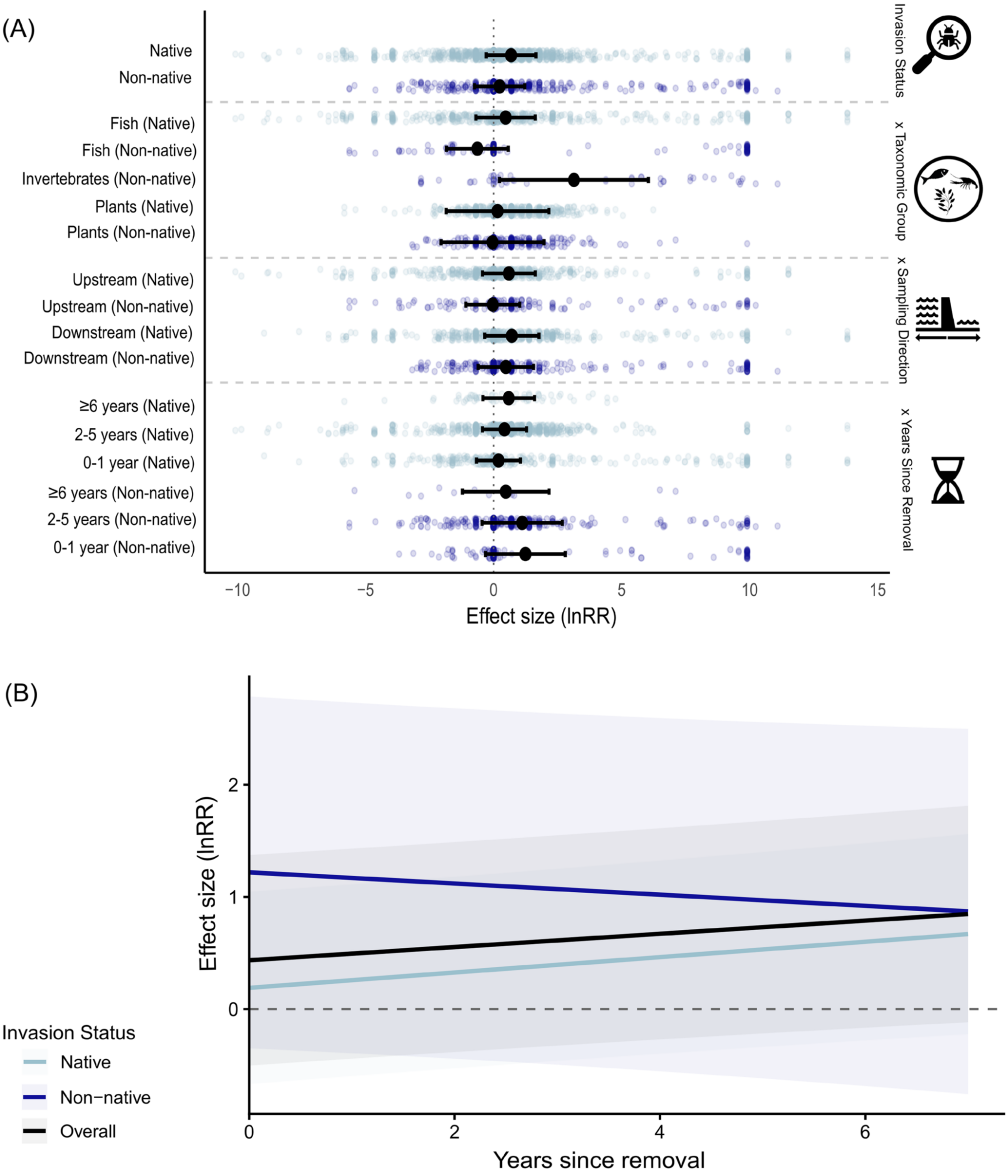
The mean estimate and 95% confidence intervals of (A) the main effect of invasion status, the interaction between invasion status and taxonomic group, sampling direction relative to the barrier, and binned years since removal. (B) Linear predicted slopes from the meta‐analysis model for each invasion status over the 7 years following barrier removal, with shaded ribbons representing 95% confidence intervals.

We found a significant effect of invasion status on populations (QM [df = 2] = 18.90, *p* < 0.001) (Figure 6a; Table S5). Both native species (*β*_native_ = 0.69, CI [−0.29, 1.66]) and non‐native species (*β*_nonnative_ = 0.23, CI [−0.75, 1.22]) exhibited positive mean effect sizes, indicating that their populations increased over time following removal; however, neither differed significantly from zero. Contrasts, however, revealed that native species exhibited significantly larger effects than non‐native species (*β*_native‐nonnative_ = 0.45, ±0.107, Wald *χ* ^2^₁ = 17.634, *p* < 0.001).

### 3.3 Responses Among Taxonomic Groups

Native and non‐native species responded differently depending on the taxonomic group (QM(5) = 39.837, *p* < 0.001; Figure 6a; Table S5). Native fish showed a tendency to increase, while non‐native fish showed a tendency to decrease following barrier removal (*β*_native_ = 0.469, CI [−0.691, 1.628]; *β*_nonnative_ = −0.634, CI [−1.838, 0.57]). While there were no native invertebrates in our database, non‐native invertebrates showed a significant increase in populations (*β*_nonnative_ = 3.138, CI[0.231, 6.045]). Native plants showed a non‐significant increase and non‐native populations decreased following barrier removals (*β*_native_ = 0.154, CI [−1.852, 2.159]; *β*_nonnative_ = −0.043, CI [−2.052, 1.966]). Contrasts showed that native populations of fish had significantly more positive responses than non‐native populations (*β*_native‐nonnative_ = 1.103 ± 0.193, Wald *χ* ^2^₁ = 32.621, *p* < 0.001). There was no difference between native and non‐native plants (0.196, ±0.126, Wald *χ* ^2^₁ = 2.438, *p* = 0.118).

### 3.4 Responses Upstream and Downstream of Removals

Invasion status and sampling direction had a strong overall effect, jointly explaining substantial heterogeneity (QM (3) = 24.616, *p* < 0.001; Figure 6a; Table S5). While non‐significant, both downstream (*β*_native_ = 0.711, CI[−0.35, 1.77]) and upstream (*β*_native_ = 0.597, CI[−0.436, 1.629]) from the barrier, native populations tended to respond positively. In comparison, the non‐native population tended to increase downstream (*β*_non‐native_ = 0.479, CI[−0.602, 1.56]) and decrease (*β*_non‐native_ = −0.031, CI[−1.083, 1.021]) upstream. Contrasts showed that there was no significant difference in effect sizes of native and non‐native populations downstream of the barrier (*β*_native‐nonnative_ = 0.127 ± 0.082, Wald *χ* ^2^₁ = 2.369, *p* = 0.124). Upstream, native species had significantly greater effect sizes than non‐native species (*β*_native‐nonnative_ = 0.766 ± 0.056, Wald *χ* ^2^₁ = 186.06, *p* < 0.001).

### 3.5 Temporal Divergence of Native and Non‐Native Responses

Effect sizes increased significantly over time (*β* = 0.059, CI [0.014, 0.104]) across all species, regardless of invasion status, indicating that the general strength of population growth increased with time since removal (Figure 6b; Table S5). However, when the data were separated by invasion status, native species showed a significant positive relationship between years since removal and effect size (*β*_native_ = 0.068, CI [0.02, 0.117]), whereas non‐native species showed no significant temporal trend (*β*_nonnative_ = −0.050, CI [−0.137, 0.038]), with a tendency for their positive population growth rate to decline over time, while remaining positive overall.

For native species, mean effect sizes in each time bin also did not differ from zero (*β*_0–1year_ = 0.19, CI[−0.67, 1.05]; *β*_2–5year_ = 0.43, CI[−0.44, 1.29]; *β*_≥6year_ = 0.59, CI [−0.42, 1.60]). The temporal moderator explained a small but significant portion of the overall variation (QM (2) = 6.87, *p* = 0.032), with the highest estimated mean effect size in the ≥ 6‐year bin, indicating that population growth was strongest after the longest time bin. Contrasts showed that effect sizes in the 2–5 year bin were significantly higher than the 0–1 year bin (*β* = 0.234 ± 0.101; Wald *χ* ^2^₁ = 5.423, *p* = 0.02). Again, effect sizes were greater ≥ 6 years than that of the 0–1 year bin (*β* = 0.399 ± 0.291; Wald *χ* ^2^₁ = 1.875, *p* = 0.171). There was no difference between the 2–5 and ≥ 6 year bins (*β* = 0.165 ± 0.301; Wald *χ* ^2^₁ = 0.3, *p* = 0.584).

Regarding non‐native species, there was no overall evidence that effect sizes differed across bins (QM = 5.97, *p* = 0.113), and all bin‐specific estimates overlapped zero (*β*_0–1year_ = 1.24, CI [−0.314, 2.802]; *β*_2–5year_ = 1.12, CI [−0.45, 2.682]; *β*_≥6year_ = 0.475, CI[−1.216, 2.165]). While population changes between 0–1 and 2–5 years were not significantly different (*β* = 0.128 ± 0.187; Wald *χ* ^2^₁ = 0.467, *p* = 0.494) and 2–5 compared to ≥ 6 years was marginally non‐significant (*β* = 0.64 ± 0.386; Wald *χ* ^2^₁ = 2.768, *p* = 0.096), the effect size at ≥ 6 years was significantly lower than 0–1 years (*β* = 0.77 ± 0.390; Wald *χ* ^2^₁ = 3.90, *p* = 0.048).

## 4 Discussion

Knowledge on population responses to barrier removals is strongly shaped by geographic, taxonomic and study design imbalances. Publications are heavily concentrated in North America, particularly the US, reflecting regional long‐standing investment in river restoration, environmental monitoring and academic research infrastructure (Grill et al. 2019; Lehner et al. 2024). The complete absence of studies from Africa and South America and few studies from Asia likely indicates a publication imbalance within ecological research towards the Global North, rather than the complete absence of barrier removals (Nuñez et al. 2019). This may also reflect that barrier constructions in Africa, Asia and South America have been rapidly increasing since the late 1900s and, despite some successful efforts among environmental and human rights movements, these still far outweigh removals (Dolan et al. 2025). In comparison, in North America and Europe, construction has plateaued, and rates of removals have rapidly increased (Dolan et al. 2025; Lehner et al. 2024). Nevertheless, in Europe the number of publications are still limited, creating a severe information disparity (Ding et al. 2019; Nuñez et al. 2019).

Our synthesis, which found substantial regional unevenness, may in part be attributed to the language and type of articles included. For example, the responses of riverine biota to the removal of a single barrier might have been missed by our searches due to only being documented in environmental assessment reports published in local languages but not included in peer‐reviewed papers published in English. We note that whether publications result from a barrier removal project depends on numerous factors, including the availability of funding, public and scientific engagement and the frequency to which removals occur, since they can be costly and not a priority management action for conservation considering particular aspects of these regions. Thus, these geographical trends may not demonstrate true biases in the number of removals. Nevertheless, the persistence of these imbalances evidenced in this review creates a consequential knowledge gap for effective policy implementation.

Taxonomic imbalance towards fish is consistent with their socio‐economic importance and central role in motivating barrier removals (Chan et al. 2025; He et al. 2021), but it constrains our understanding of whole‐community responses. Invertebrates are key drivers of ecosystem processes such as nutrient cycling and secondary production in river ecosystems, and their underrepresentation limits the ecological resolution of current syntheses (Panagiotou et al. 2022). The limited representation of native and non‐native invertebrates (especially insects) in barrier removal response studies potentially reflects challenges, including (i) difficulties in taxonomic identification of invertebrates, especially during larval life stages (Pires et al. 2021), and (ii) the relatively high dispersal capacity of many lotic insects during their aerial adult stage, which may reduce the detectability of localised responses to barrier removal within specific river sections (Bohonak and Jenkins 2003). However, the dispersal of non‐insect freshwater invertebrates, such as crustaceans and molluscs (including the three non‐native invertebrates identified in our study), is of the most concern for their biological invasions (Fenoglio et al. 2016). In addition, the responses of benthic algae, essential primary producers in river ecosystems that are sensitive to barrier‐induced environmental changes (Wu et al. 2009; Couto and Olden 2018), have rarely been investigated. While community composition, trophic networks and ecosystem functioning are changed by long‐term abiotic conditions, their responses to barrier removals have yet to be synthesised in published studies (Cai et al. 2024; Tonkin, Altermatt, et al. 2018).

Peer‐reviewed literature remains disproportionately focused on dam removals (Bellmore et al. 2017), probably reflecting the interest in large dams due to the scale of ecological impacts or socio‐political provisions, and thus funding availability. Also, inconsistent language and quantitative thresholds used to describe riverine barriers within ecological studies and environmental policies could contribute to such imbalance. From our synthesis, the term ‘dam’ was used to describe structures ranging in height (ranging from 1.5 m to 36 m) and design, though many authors provided very few quantitative details on their dimensions. Several studies have shown that the cumulative effects of multiple small barriers in a catchment can be similar to or more detrimental than those of a single large barrier (Li et al. 2021; Morden et al. 2022), highlighting the importance of reporting barriers structural characteristics in studies. Removal responses of low‐head barriers compared to large dams are likely to be different at broad ecological scales due to barrier characteristics (e.g., flooded area, size of the structure, time since construction), and the spatial–temporal scales of restoration (Brown et al. 2024; Lepš et al. 2016). However, comparison between ecosystem‐level responses and various barrier types remains critically understudied. The persistence of a dam‐centric evidence base can have serious implications, for example, by amplifying disconnection between scientific research focus and management investments. This will become more evident considering that restoration efforts are increasingly removing smaller structures (Mouchlianitis 2026).

Our meta‐analysis informs the isolation‐invasion dilemma that challenges river restoration, by quantitatively discerning native and non‐native population responses for the first time (Fausch et al. 2009; Rahel and McLaughlin 2018). Considering the overall results across all taxa, population sizes tended to increase following barrier removal, but native species exhibited significantly stronger positive responses than non‐native species. This finding provides quantitative support for the idea that restoring connectivity could preferentially benefit native biota. Moreover, we further highlight spatial, temporal and taxonomic context‐dependencies that alter responses between native and non‐native species populations. The results from these analyses should be carefully considered, since quantitative analyses were based on only available, published data on the topic, and may reflect a lack of research rather than true ecological responses, and results are only of already established non‐native populations pre‐removal. Whereas, the new occurrence of additional non‐native species following barrier removals is a substantial concern, given that their populations can rapidly grow with subsequent potential for impact (Nordheimer and Jeschke 2018).

Upstream of removed barriers, native populations responded more positively than non‐native populations, whereas downstream differences were weaker. This suggests that reconnecting historically isolated habitats may primarily enhance recolonisation and demographic recovery of native species, without necessarily promoting upstream expansion of non‐native taxa. These results are likely to be further mediated by context‐dependencies shaped by habitat suitability, legacy invasion pressure and biotic resistance (Jones et al. 2020; Dolan et al. 2025). Moreover, given that invasions in freshwater systems can be subject to time lags before rapid population and impact increases, often lasting decades, the short duration of studies included in our review may mask the longer‐term implications for invasion dynamics upstream after connectivity is restored (Soto et al. 2023). Continued introductions and spread of non‐native species through expanding trade and transport networks may further compound these delayed responses, creating additional uncertainty surrounding future invasion risks following barrier removal (Bray et al. 2024; Britton et al. 2023).

Despite some contrasting responses among taxonomic groups (e.g., native versus non‐native fish), our results generally support the long‐standing focus of barrier removal on restoring migratory pathways for native fishes. However, our findings may simply reflect specific functions and traits, particularly mechanisms of dispersal, of a few successful native species, such as migratory, large‐bodied fish that readily exploit newly connected systems (Höckendorff et al. 2017). Future research is required to explore how inter‐ and intraspecific life history traits may lead to variations in population recoveries. Populations which have been extirpated by the presence of a barrier may require longer time to recover, whereas those populations for which human intervention aided species recovery, response rates may increase. In contrast, apparent declines in some non‐native fish populations following barrier removal may not necessarily indicate population reduction but instead reflect the redistribution or diffusion of individuals into newly accessible reaches upstream of former barriers. Consequently, studies focused only on localised reaches adjacent to barriers or short‐term temporal responses may fail to capture broader basin‐scale population dynamics.

The significant increase detected for non‐native invertebrates highlights a potential blind spot within the rapid expansion of connectivity restoration. Once established, non‐native invertebrates can negatively affect native species through competition, predation and ecosystem modification. For example, the New Zealand mudsnail and virile crayfish, two invertebrate species in our meta‐analysis which significantly increased following barrier removals, outcompete native invertebrates for food provisions, alter predation pressures and influence space by modifying habitat complexity (Alonso and Castro‐Díez 2008; Miller et al. 2024). Once introduced, the New Zealand mudsnail can dominate entire river systems (Haubrock et al. 2022), contributing to most of the invertebrate secondary production, effectively sequestering carbon and restructuring food‐web function (Hall Jr. et al. 2006). North American crayfish species, including virile crayfish, have demonstrated ecological effects (Twardochleb et al. 2013). Artificial barriers have often reduced the upstream spread of non‐native crayfish, which protected native crayfish, while in comparative free‐flowing rivers, native crayfish went extinct because of the encroachment from non‐native crayfish (Chucholl et al. 2022). These cases exemplify how non‐native invertebrates can exploit better‐connected river systems following barrier removals.

The number of years since removal mediates population responses, evidencing the importance of long‐term and large‐scale perspectives in evaluating restoration success. Short monitoring windows may underestimate native recovery while failing to detect delayed responses among non‐native species. Biological invasions often show a time lag in their detection and impact, where demographic and community‐level responses can unfold over decades (Duncan 2021; Robeck et al. 2024; Spear et al. 2021); such responses could not be captured in our analysis due to limited study time spans. Without longer‐term data, it remains difficult to assess whether observed trends persist, stabilise or reverse over time.

Improving clarity and consistency in invasion terminology is also essential for advancing synthesis and informing management. Our focus on the invasion status of species, through targeted searches on non‐native versus native species responses, may have excluded more general restoration studies or the ones focused solely on endemic or native species. This reflects a broader issue in ecology around inconsistent identification and reporting of native and non‐native status (Soto et al. 2024) that can obscure impacts of non‐native species and ultimately jeopardise conservation actions. Despite efforts to standardise language (Groom et al. 2019; Richardson et al. 2010), a consistent rubric has yet to be adopted. Addressing this issue will require harmonisation of terminology across publications, adopting a simplified framework that allows for clear definition of populations and explicitly defining the term(s) when they are applied by researchers, managers and policymakers (see Soto et al. 2024).

## 5 Future Directions to Resolve Research Imbalances and Improve Conservation Outcomes

**FIGURE 7:**
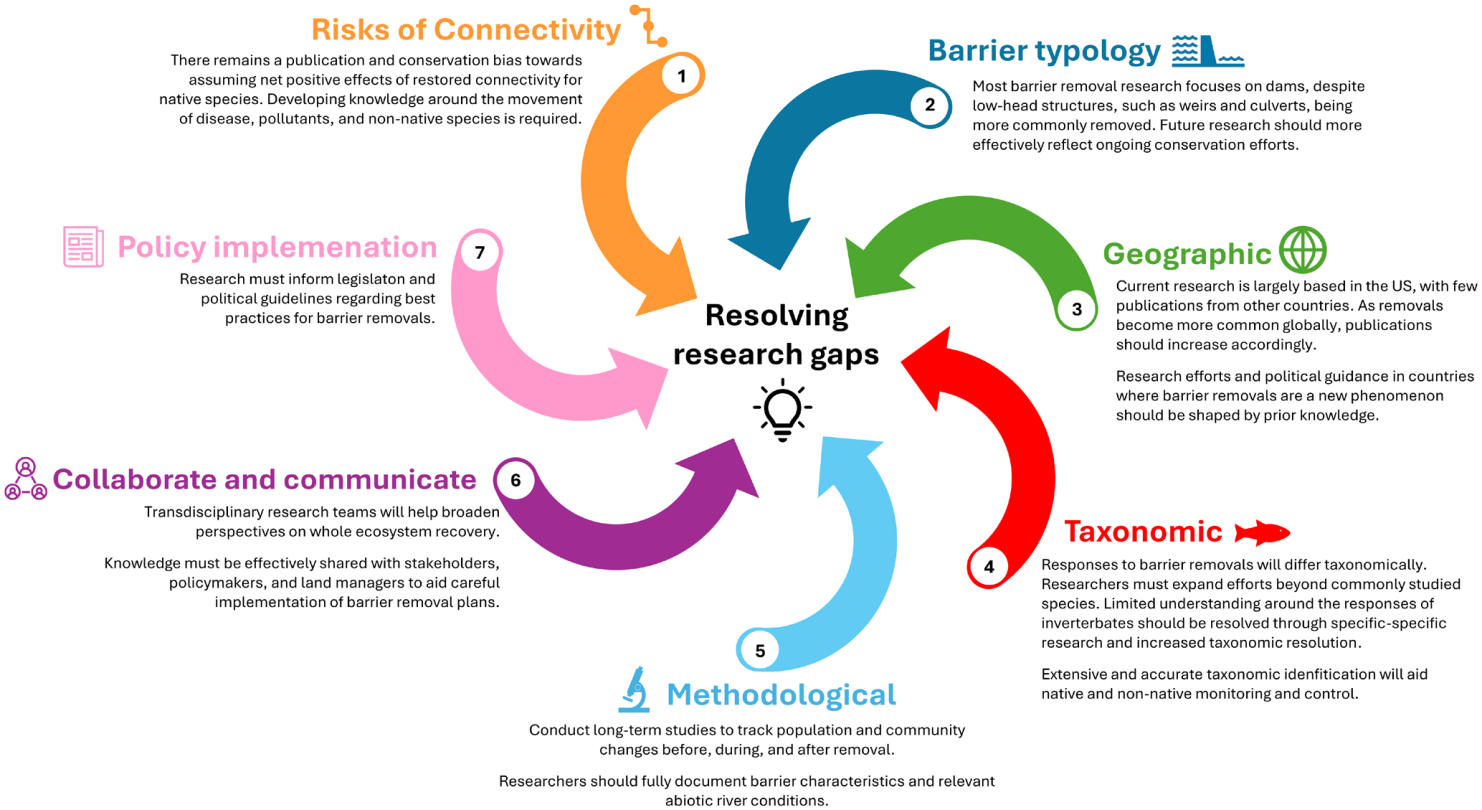
Recommended future directions of research regarding ecological responses to riverine barrier removals.

As barrier removals accelerate under ambitious connectivity restoration agendas (e.g., see European Commission 2021), our findings provide cautious but encouraging evidence that native populations often respond positively to barrier removals. However, promotion of non‐native species populations can also concurrently occur, albeit at a slower rate. Therefore, the native population growth pattern should not be interpreted as evidence that connectivity restoration is consistently low risk. Considering potential perverse outcomes, including the spread of existing and potential non‐native species, is particularly vital as restoration of river connectivity intensifies globally (Firth et al. 2025). Failure to explicitly account for these risks may result in conservation actions that inadvertently undermine biodiversity objectives. We suggest several next steps to align research with the pace and scale of conservation policies and actions, ensuring that barrier removals deliver sustainable ecological benefits (Figure 7).

A central research priority is the development of long‐term, standardised monitoring frameworks, beyond the typical short post‐removal assessment period of less than five years. We suggest that intentional planning for long‐term monitoring of riverine populations and communities should be a collaborative endeavour by independent conservation groups and research institutes, including ascertaining appropriate biological baseline data. Extended monitoring programmes are crucial for capturing full ecosystem recovery and identifying any delays or non‐linear responses. Biological responses from native and non‐native species to river restoration are highly species‐specific through time, with both short‐ and long‐term responses depending on species' characteristics (Haubrock et al. 2022; Höckendorff et al. 2017; Spear et al. 2021). Thus, a short‐term focus on initial hydrological recovery post‐removal could miss unanticipated chronic changes to ecosystems.

Broadening research across barrier typologies is of the utmost importance. Low‐head barriers outnumber large dams in most rivers globally (Couto and Olden 2018), but removal focuses differ regionally, with barrier removal policies within the EU being particularly focused on low‐head structures, such as weirs and culverts. While many projects in the US have been large‐scale, costly dam removals, low‐head barrier removals have been less reported on by media yet remain common (Belletti et al. 2020; Bellmore et al. 2017). Considering the physical and structural design differences within typologies (e.g., material, height and crest width), as well as the presence or absence of a fish passage, may also substantially change the scale to which native and non‐native movements are impeded. Due to varying characteristics among barriers, even within types, the effects on populations differ, especially under the context of flood events which may increase ability of motile species to pass, particularly over low‐head barriers (Aleyasin et al. 2024).

In this complex scenario, global restoration targets cannot be allowed to be based on a foundation of published and peer reviewed evidence derived largely from North America, mirroring broader biases in ecological research (Bol et al. 2023). While barrier removals have thus far occurred predominantly within the US, there remains a need for greater research effort in other regions to establish baseline understandings of riverine communities and their responses to barrier removal. Expanding ecological monitoring across diverse geographical, social and economic contexts will improve understanding of restoration outcomes and support the development of locally relevant conservation strategies. Targeted research is required to develop well‐informed risk prioritisation frameworks guided by rigorous ecological understanding aligned with the affected local community.

Another major research concern is the restricted taxonomic scope. The most commonly studied species are those already prioritised in river conservation programmes, typically migratory fishes, while the responses of algae, plants, invertebrates, amphibians, birds, reptiles, mammals and many less charismatic resident fish species to barrier removal remain understudied. Given the widely documented barrier impacts on non‐fish species in rivers (Altanov et al. 2025; Dare et al. 2020; Wu et al. 2019), this narrow lens restricts our ability to evaluate whole‐ecosystem consequences and may obscure important cross‐taxonomic interactions following barrier removal. In particular, there is a frequent lack of species‐level resolution, with many studies, especially for invertebrates at larval stage, reporting responses at coarse taxonomic groupings (e.g., genus or family). This constrains our ability to determine whether barrier removals yield positive or negative outcomes for native and non‐native species. It is essential that research is conducted at more granular taxonomic resolutions so that conservation gains can be distinguished from unintended ecological consequences. Functional and life history traits within taxonomic groups, such as the mode of locomotion among motile species, are important to consider under the context of the abiotic factors. For example, invasive crayfish species which can exploit their swimming or walking abilities to disperse, can make barriers futile in slowing their spread, but also mean that they can readily take advantage of newly exposed habitats after barrier removals (Thomas et al. 2019). Thus, an umbrella conclusion for all barrier removal plans will be ineffective; rather, we recommend project‐specific consideration of the risks of barrier removal. In addition, those that have identified taxa to species level have often adopted a narrow focus on either native or non‐native taxa, rather than intentionally evaluating responses across both groups simultaneously. Adopting a broader ecological perspective, which incorporates both native and non‐native species and examines community‐level and ecosystem‐level changes, is critical for identifying indirect and cascading effects of restored connectivity (Carneiro et al. 2025).

Details of study design, such as duration of study, but also direction and distance of sampling, can strongly shape the concluding results of studies regarding population recovery, recolonisation potential and invasion risk. Directionality is of particular interest for controlling non‐native species, which can differ intraspecifically and interspecifically in their mechanisms and success of dispersal (Osawa et al. 2013; Wagner et al. 2020). Informed reporting of these details can facilitate inference of behavioural ecology and incorporating spatial movement data into future analyses will enhance predictions of which species are likely to benefit or disbenefit from restored connectivity.

With comprehensive planning, new constructions can have a low environmental impact. For example, the concept of ‘smart’ siting of new dams has gained popularity, utilising Geographic Information Systems and remote sensing tools to find locations for dam building that minimise the negative impacts on native biodiversity and protect natural habitats (Couto et al. 2021). To balance socio‐economic demands with ecological necessity, as well as to remove barriers with hazardous status due to age, modifying barriers rather than completely deconstructing them is also a potential solution (Dolan et al. 2025; Ho et al. 2017; McKay et al. 2020). Already underway in many regions, retrofitting or replacing barriers can be successful; however, many knowledge gaps remain (Zubick et al. 2025). For example, the addition of baffles to change water velocity through culverts at road crossings can provide more suitable conditions for fish passage. Fixing bristle boards or studs to weir surfaces can also aid eel and sea lamprey passage (Franklin and Baker 2025; Vowles et al. 2015). However, these passages can introduce other challenges, as non‐native species may also exploit infrastructure designed for native species (Welsh and Loughman 2015).

Policy frameworks for connectivity restoration must be prepared for unforeseen, negative outcomes following barrier removals, particularly concerning new or unrecorded invasive species in the river network. As river networks become increasingly invaded, decision‐making must explicitly weigh the ecological benefits of reconnection against the risk of facilitating non‐native species spread (Haubrock et al. 2023; Seebens et al. 2017). Effective implementation will require risk‐based, spatially explicit prioritisation of barrier removals, informed by invasion history, species traits and conservation value of habitats. In some systems, full removal may be inappropriate and selective retention, partial removal or structural modification may better align with conservation objectives—particularly where barriers isolate and protect threatened native populations from non‐native predators or competitors. Control of non‐native species should be consistently integrated into restoration plans (see example treatments in Haak and Williams 2013 and Marks et al. 2010), rather than serving as an *ad hoc* response. To avoid unintended outcomes and costs, mandatory pre‐ and post‐removal monitoring and adaptive management strategies should be in place, where initial grant plans include long‐term funding.

Effective conservation outcomes will require integration of local interests, land management, research, science communication and policy. Multidisciplinary research teams working on river restoration projects can provide cost‐ and energy‐efficient approaches for gathering insight on the ecosystem‐wide success of these efforts. Simultaneously, these teams can provide experienced advice for future projects and policies which cover the scope of multiple global stressors, including biological invasions and climate change. Improved communication across disciplinary silos, along with greater transparency and data sharing, would accelerate learning and reduce duplicated efforts.

The vast majority of barrier removal programmes are conducted by independent conservation and engineering agencies and non‐government organisations, which have successfully monitored and managed the responses of the aquatic communities. However, publication of these data and associated reports is uncommon. We thus encourage organisations to publish their work using open science platforms and scientific communications teams to promote them. Embedding the results from rigorous, long‐term scientific monitoring of ecosystem responses to barrier removals into policy frameworks for integrated basin management, particularly in the context of adding or removing multiple stressors in rivers, will help create synergistically beneficial outcomes for biodiversity conservation at basin scale. These long‐term projects can be efficiently conducted through targeted research based on horizon scanning of biological invasions, with field studies further supported by regular reporting from citizen science and local or Indigenous community records and experiences (Austen et al. 2024; Jessen et al. 2022).

## 6 Conclusions

With continued efforts to remove artificial barriers for native species movement, minimising the potential risk of non‐native species spread is vital. We have harmonised the peer‐reviewed research to date on population recovery after barrier removals. We highlight that there remains a paucity of knowledge, both geographically and temporally, as well as taxonomically and methodologically, regarding how populations may respond to the removal of artificial barriers. Although the issue is complex, negative outcomes can be minimised if researchers, policymakers and conservation practitioners incorporate the effects of multiple stressors into restoration plans. By broadening research focus, improving study design, acknowledging ecological trade‐offs and strengthening interdisciplinary collaboration, future efforts can more effectively balance the benefits and risks of barrier removal and ultimately enhance conservation outcomes.

## Author Contributions

**Fengzhi He:** writing – review and editing. **Ismael Soto:** investigation, writing – review and editing, formal analysis, visualization, methodology. **Ross N. Cuthbert:** supervision, conceptualization, writing – review and editing, investigation, methodology. **Julian D. Olden:** writing – review and editing. **Jonathan D. Tonkin:** writing – review and editing. **Ellen J. Dolan:** conceptualization, investigation, writing – original draft, methodology, validation, visualization, formal analysis. **Jaimie T. A. Dick:** writing – review and editing, supervision, conceptualization, methodology. **Laís Carneiro:** writing – review and editing, investigation.

## Funding

This work was supported by the Department of Agriculture, Environment and Rural Affairs, UK Government. Excelencia Severo Ochoa grant (CEX2024‐001498‐S) funded by MICIU/AEI/10.13039/501100011033. Rutherford Discovery Fellowship, RDF‐18‐UOC‐007. Richard C. and Lois M. Worthington Endowed Professor. Ministry of Business, Innovation and Employment, CAWX2101. Chinese Academy of Sciences, E355S122, E529S101.

## Conflicts of Interest

The authors declare no conflicts of interest.

## Supporting information


**Table S1:** Boolean search string combinations.
**Table S2:** Inclusion and exclusion criteria used to compile the dataset for the systematic review and meta‐analysis.
**Table S3:** Publications included in the systematic review and meta‐analysis.
**Table S4:** The list of descriptors in the respective systematic and meta‐analysis datasets used in the present study. All data are available at the Zenodo repository in the data availability statement.
**Table S5:** Summary of multivariate meta‐analytic models (REML; *k* = 2840). Significant results are in bold.
**Figure S1:** PRISMA diagram of paper inclusion and exclusion for final systematic review and meta‐analysis.

## Data Availability

The data that support this article are available in Zenodo (doi: 10.5281/zenodo.20280649).
