# Supplementary material for: Native and Non‐Native Populations Respond Unevenly to River Barrier Removals
Source: Glob Chang Biol. 2026 Jun 8;32(6):e70941. doi: 10.1111/gcb.70941 (PMC13244418; doi:10.1111/gcb.70941)
Supplement: Supplementary file 1 — Table S1: Boolean search string combinations. Table S2: Inclusion and exclusion criteria used to compile the dataset for the systematic review and meta‐analysis. Table S3: Publications included in the systematic review and meta‐analysis. Table S4: The list of descriptors in the respective systematic and meta‐analysis datasets used in the present study. All data are available at the Zenodo repository in the data availability statement. Table S5: Summary of multivariate meta‐analytic models (REML; k = 2840). Significant results are in bold. Figure S1: PRISMA diagram of paper inclusion and exclusion for final systematic review and meta‐analysis. [file GCB-32-e70941-s001.docx]

**Supplementary information**

**Table S1.** Boolean search string combinations.

| Labels | | Search string | Number of results (WoS all databases) |
| --- | --- | --- | --- |
| 1 | Always included | ALL=(dam* OR weir* OR impoundment* OR barrier* OR obstacle* OR reservoir* OR sluice* OR culvert* OR ford* OR ramp*) AND ALL=(remov* OR deconstruct* OR destruct* OR undam*) AND ALL=(river* OR stream* OR fluvial* OR freshwater* OR lotic*) | - |
| 2 | Native search string added to (1) | AND ALL=(native* OR indigenous OR endemic OR "local species" OR autochthonous) | 738 |
| 3 | Non-native search string added to (1) | AND ALL=(invas* OR "non-native*" OR "nonindigenous" OR "non indigenous" OR "alien species" OR exotic OR "introduced species" OR nonnative) | 1,147 |

**Table S2.** Inclusion and exclusion criteria used to compile the dataset for the systematic review and meta-analysis.

| Review stage | Inclusion | | Exclusion | |
| --- | --- | --- | --- | --- |
| Systematic | Reason 1 | Assessed the influence of the removal (intentional or unintentional; complete or incomplete) of an anthropogenic barrier (of any design). | Reason 1 | Study not related to anthropogenic barriers. |
|  |  |  | Reason 2 | No barrier removal. |
|  |  |  | Reason 3 | No primary source material (e.g. opinion piece, narrative review) |
|  | Reason 2 | Assessed any taxonomic group within the watershed where the barrier is/was present. | Reason 4 | Authors produced a removal framework or used predictive modelling of population response. |
|  |  |  | Reason 5 | No measurement of biotic population. |
| Meta-analysis | Reason 3 | Publications were included when statistical measures were of population size were provided, even of variability of the measure (e.g. SD or SE) were not. | Reason 6 | No population measurements from both before and after. |
|  |  |  | Reason 7 | Classification to species level is not provided and thus, invasion status cannot be deciphered. |

**Table S3.** Publications included in the systematic review and meta-analysis.

| Number | Authors | Title | Year | Journal | DOI | Included in meta-analysis |
| --- | --- | --- | --- | --- | --- | --- |
| 1 | Collins, SF; Marshall, B; Moerke, A | Aerial insect responses to non-native Chinook salmon spawning in a Great Lakes tributary | 2016 | Journal of Great Lakes Research | 10.1016/j.jglr.2016.02.010 | N |
| 2 | Adams, KJ; Marks, JC | Population response of the invasive crayfish Orconectes virilis (Hagen, 1870) (Decapoda: Astacoidea: Cambaridae) to restoration: What are the consequences of changes in predatory regulation and physical habitat in Fossil Creek, Arizona, USA? | 2016 | Journal of Crustacean Biology | 10.1163/1937240X-00002471 | Y |
| 3 | Shaffer, JA; Juanes, F; Quinn, TP; Parks, D; McBride, T; Michel, J; Naumann, C; Hocking, M; Byrnes, C | Nearshore fish community responses to large scale dam removal: implications for watershed restoration and fish management | 2017 | Aquatic Sciences | 10.1007/s00027-017-0526-3 | Y |
| 4 | Marks, JC; Haden, GA; O'Neill, M; Pace, C | Effects of Flow Restoration and Exotic Species Removal on Recovery of Native Fish: Lessons from a Dam Decommissioning | 2010 | Journal of the Society for Ecological Restoration | 10.1111/j.1526-100X.2009.00574.x | Y |
| 5 | Ravot, C; Laslier, M; Hubert-Moy, L; Dufour, S; Le Coeur, D; Bernez, I | Large dam removal and early spontaneous riparian vegetation recruitment on alluvium in a former reservoir: Lessons learned from the pre-removal phase of the Selune River project (France) | 2020 | River Research and Applications | 10.1002/rra.3535 | N |
| 6 | Stanley, EH; Catalano, MJ; Mercado-Silva, N; Orra, CH | Effects of dam removal on brook trout in a Wisconsin stream | 2007 | River Research and Applications | 10.1002/rra.1021 | Y |
| 7 | Prach, K; Chenoweth, J; del Moral, R | Spontaneous and assisted restoration of vegetation on the bottom of a former water reservoir, the Elwha River, Olympic National Park, WA, USA | 2019 | Restoration Ecology | 10.1111/rec.12915 | N |
| 8 | Lisius, GL; Snyder, NP; Collins, MJ | Vegetation community response to hydrologic and geomorphic changes following dam removal | 2018 | River Research and Applications | 10.1002/rra.3261 | Y |
| 9 | Ding, CZ; Jiang, XM; Wang, LE; Fan, H; Chen, LQ; Hu, JM; Wang, HL; Chen, YF; Shi, XT; Chen, H; Pan, BH; Ding, LY; Zhang, C; He, DM | Fish Assemblage Responses to a Low-head Dam Removal in the Lancang River | 2019 | Chinese Geographical Science | 10.1007/s11769-018-0995-x | Y |
| 10 | Orr, CH; Stanley, EH | Vegetation development and restoration potential of drained reservoirs following dam removal in Wisconsin | 2006 | River Research and Applications | 10.1002/rra.891 | N |
| 11 | Cook, KL; Wallender, WW; Bledsoe, CS; Pasternack, G; Upadhyaya, SK | Effects of Native Plant Species, Mycorrhizal Inoculum, and Mulch on Restoration of Reservoir Sediment Following Dam Removal, Elwha River, Olympic Peninsula, Washington | 2011 | Restoration Ecology | 10.1111/j.1526-100X.2009.00559.x | N |
| 12 | Brenkman, SJ; Mumford, SL; House, M; Patterson, C | Establishing baseline information on the geographic distribution of fish pathogens endemic in Pacific salmonids prior to dam removal and subsequent recolonization by anadromous fish in the Elwha River, Washington | 2008 | Northwest Science | 10.3955/0029-344X-82.S.I.142 | N |
| 13 | Magilligan, FJ; Nislow, KH; Kynard, BE; Hackman, AM | Immediate changes in stream channel geomorphology, aquatic habitat, and fish assemblages following dam removal in a small upland catchment | 2016 | Geomorphology | 10.1016/j.geomorph.2015.07.027 | Y |
| 14 | Raabe, JK; Hightower, JE | Assessing Distribution of Migratory Fishes and Connectivity following Complete and Partial Dam Removals in a North Carolina River | 2014 | North American Journal of Fisheries Management | 10.1080/02755947.2014.938140 | N |
| 15 | Gillette, DP; Daniel, K; Redd, C | Fish and Benthic Macroinvertebrate Assemblage Response to Removal of a Partially Breached Low-head Dam | 2016 | River Research and Applications | 10.1002/rra.3017 | N |
| 16 | Cubley, ES; Brown, RL | Restoration of Hydrochory Following Dam Removal on the Elwha River, Washington | 2016 | River Research and Applications | 10.1002/rra.2999 | Y |
| 17 | Villamil, JJC; Locke, SA | Fish assemblage response to removal of a low-head dam in the lower reach of a tropical island river | 2022 | Freshwater Biology | 10.1111/fwb.13893 | Y |
| 18 | Lake, TRT; Ravana, KR; Saunders, R | Evaluating Changes in Diadromous Species Distributions and Habitat Accessibility following the Penobscot River Restoration Project | 2012 | Marine and Coastal Fisheries | 10.1080/19425120.2012.675971 | N |
| 19 | Chenoweth, J; Bakker, JD; Acker, SA | Planting, seeding, and sediment impact restoration success following dam removal | 2022 | Restoration Ecology | 10.1111/rec.13506 | N |
| 20 | Brown, RL; Thomas, CC; Cubley, ES; Clausen, AJ; Shafroth, PB | Does large dam removal restore downstream riparian vegetation diversity? Testing predictions on the Elwha River, Washington, USA | 2022 | Ecological Applications | 10.1002/eap.2591 | Y |
| 21 | Mahan, DC; Betts, JT; Nord, E; Van Dyke, F; Outcalt, JM | Response of benthic macroinvertebrates to dam removal in the restoration of the Boardman River, Michigan, USA | 2021 | PLoS ONE | 10.1371/journal.pone.0245030 | Y |
| 22 | Sun, JR; Tummers, JS; Galib, SM; Lucas, MC | Fish community and abundance response to improved connectivity and more natural hydromorphology in a post-industrial subcatchment | 2022 | Science of the Total Environment | 10.1016/j.scitotenv.2021.149720 | Y |
| 23 | Scoppettone, GG; Rissler, PH; Nielsen, MB; Harvey, JE | The status of Moapa coriacea and Gila seminuda and status information on other fishes of the Muddy River, Clark County, Nevada | 1998 | The Southwestern Naturalist | (DOI not provided) | Y |
| 24 | Thomson, HM; Davies, MM; Lawn, PTES; Kushneryk, K; Brouard-John, EK; Nelson, KR; Gerwing, TG | Spread of an Aquatic Invasive Plant, Iris pseudacorus, Following Replacement of a Hydraulic Structure | 2021 | Ecological Restoration | 10.3368/er.39.4.238 | Y |
| 25 | Chung, LC; Lin, HJ; Yo, SP; Tzeng, CS; Yeh, CH; Yang, CH | Relationship between the Formosan landlocked salmon Oncorhynchus masou formosanus population and the physical substrate of its habitat after partial dam removal from Kaoshan Stream, Taiwan | 2008 | Zoological Studies | (DOI not provided) | Y |
| 26 | Livermore, J; Trainor, M; Bednarski, MS | Successful Spawning of Anadromous Petromyzon marinus L. (Sea Lamprey) in a Restored Stream Channel Following Dam Removal | 2017 | Northwestern Naturalist | 10.1656/045.024.0306 | N |
| 27 | Thoni, R; Holcomb, J; Nichols, R; Gangloff, MM | Effects of Small Dams on Sunfish Assemblages in North Carolina Piedmont and Coastal Plain Streams | 2013 | Transactions of the American Fisheries Society | 10.1080/00028487.2013.829125 | N |
| 28 | Gangloff, MM; Hartfield, EE; Werneke, DC; Feminella, JW | Associations between small dams and mollusk assemblages in Alabama streams | 2011 | Journal of North American Benthological Studies | 10.1899/10-092.1 | N |
| 29 | Peterson, DP; Neville, HM | Comparison of Methods to Verify Upstream Passage by Trout at Remediated Culverts in Four Rocky Mountain Streams | 2019 | North American Journal of Fisheries Management | 10.1002/nafm.10308 | N |
| 30 | Marks, JC; Parnell, R; Carter, C; Dinger, EC; Haden, GA | Interactions between geomorphology and ecosystem processes in travertine streams: Implications for decommissioning a dam on Fossil Creek, Arizona | 2006 | Geomorphology | 10.1016/j.geomorph.2006.01.008 | N |
| 31 | Grote, AB; Bailey, MM; Zydlewski, JD | Movements and Demography of Spawning American Shad in the Penobscot River, Maine, prior to dam removal | 2014 | Transactions of the American Fisheries Society | 10.1080/00028487.2013.864705 | N |
| 32 | Teichert, N; Lizé, A; Tabouret, H; Gérard, C; Bareille, G; Acou, A; Carpentier, A; Trancart, T; Virag, LS; Robin, E; Druet, M; Prod'Homme, J; Feunteun, E | A multi-approach study to reveal eel life-history traits in an obstructed catchment before dam removal | 2022 | Hydrobiologia | 10.1007/s10750-022-04833-9 | N |
| 33 | Reeves, DB; Tate, WB; Jelks, HL; Jordan, F | Response of Imperiled Okaloosa Darters to Stream Restoration | 2016 | North American Journal of Fisheries Management | 10.1080/02755947.2016.1227402 | N |
| 34 | Winans, GA; McHenry, ML; Baker, J; Elz, A; Goodbla, A; Iwamoto, E; Kuligowski, D; Miller, KM; Small, MP; Spruell, P; Van Doornik, D | Genetic inventory of anadromous Pacific salmonids of the Elwha River prior to dam removal | 2008 | Northwest Science | 10.3955/0029-344X-82.S.I.128 | N |
| 35 | Winans, GA; Allen, MB; Baker, J; Lesko, E; Shrier, F; Strobel, B; Myers, J | Dam trout: Genetic variability in Oncorhynchus mykiss above and below barriers in three Columbia River systems prior to restoring migrational access | 2018 | PLoS ONE | 10.1371/journal.pone.0197571 | Y |
| 36 | Peters, RJ; Person, JH; Duda, JJ; McHenry, M; Pess, GR; Brenkman, SJ; Johnson, JR; Liermann, MC; Denton, KP; Beirne, MM; Crain, P; Connor, HA | Challenges of implementing a multi-agency monitoring and adaptive management strategy for federally threatened Chinook salmon and steelhead trout during and after dam removal in the Elwha River | 2024 | Frontiers in Environmental Science | 10.3389/fenvs.2024.1291265 | Y |
| 37 | Kardouni, J; Lindsay, MD; Labay, A; Bauman, JM | Riverbank lupine’s (Lupinus rivularis) influence on conifer growth, ectomycorrhizal colonization, and neighboring vegetation in coarse sediments left behind after dam removal | 2023 | Frontiers in Ecology and Evolution | 10.3389/fevo.2023.1214117 | N |
| 38 | Chung, LC; Lin, HJ; Yo, SP; Tzeng, CS; Yang, CH | Stage-structured population matrix models for the Formosan landlocked salmon (Oncorhynchus masou formosanus) in Taiwan | 2007 | The Raffels Bulletin of Zoology | (DOI not provided) | Y |
| 39 | Kvach, Y; Stepien, CA; Minicheva, GG; Tkachenko, P | Biodiversity effects of the Russia-Ukraine War and the Kakhovka Dam destruction: ecological consequences and predictions for marine, estuarine, and freshwater communities in the northern Black Sea | 2025 | Ecological Processes | 10.1186/s13717-025-00577-1 | Y |
| 40 | Hurst, CN; Holt, RA; Bartholomew, JL | Dam Removal and Implications for Fish Health: Ceratomyxa shasta in the Williamson River, Oregon, USA | 2012 | North American Journal of Fisheries Management | 10.1080/02755947.2012.655843 | N |
| 41 | Jared B. Lamy, Brigid C. O'Donnell, Amy M. Villamagna, Tyson R. Morrill, Ben J. Nugent, Joshua C. Hoekwater | Genetic analysis reveals a complex mosaic of admixture in Brook Trout in a historically fragmented watershed | 2023 | North American Journal of Fisheries Management | 10.1002/nafm.10906 | N |
| 42 | Raymond, S; Koehn, J; Tonkin, Z; Todd, C; Stoessel, D; Hackett, G; O'Mahony, J ; Berry, K ; Lyon, J ; Sharley, J ; Moloney, P | Differential responses by two closely related native fishes to restoration actions | 2019 | Restoration Ecology | **10.1111/rec.13008** | Y |
| 43 | Winans, GA; Baker, J; McHenry, M; Ward, L; Myers, J | Genetic Characterization of Oncorhynchus mykiss Prior to Dam Removal with Implications for Recolonization of the Elwha River Watershed, Washington | 2017 | Transactions of the American Fisheries Society | 10.1080/00028487.2016.1249293 | N |
| 44 | Karberg, JM; Beattie, KC; O'Dell, DI; Omand, KA | Tidal Hydrology and Salinity Drives Salt Marsh Vegetation Restoration and Phragmites australis Control in New England | 2018 | Wetlands | 10.1007/s13157-018-1051-4 | N |
| 45 | Solomon, LE; Casper, AF; Maxson, KA; Lamer, JT; Ford, TW; Blodgett, KD; Hobson, T; Perry, D; Grider, NT; Hilsabeck, RB; Cook, TR; Irons, KS; McClelland, MA; O'Hara, TM | A Case Study of Large Floodplain River Restoration: Two Decades of Monitoring the Merwin Preserve and Lessons Learned through Water Level Fluctuations and Uncontrolled Reconnection to a Large River | 2022 | Wetlands | 10.1007/s13157-022-01581-3 | Y |

**Table S4.** The list of descriptors in the respective systematic and meta-analysis datasets used in the present study. All data are available at the Zonodo repository in the data availability statement.

| Column Codes | Descriptions | Included in systematic review | Included in meta-analysis |
| --- | --- | --- | --- |
| Paper_ID | Bibliographic data | Y | Y |
| URL | Bibliographic data | Y | Y |
| DOI | Bibliographic data | Y | Y |
| Paper_Title | Bibliographic data | Y | Y |
| Authors | Bibliographic data | Y | Y |
| PubYear | Bibliographic data | Y | Y |
| Country | Name of country where barrier is/was present. If this is not provided in the paper, it is devised from Google maps. | Y | Y |
| Continent | Name of continent where barrier is/was present. If this is not provided in the paper, it is devised from Google maps. | Y | Y |
| BarrierType | Must be dam, weir, sluice, culvert, ford, or ramp (names given by the EU for intended removals in freshwater systems). | Y | Y |
| BarrierType_ifother | If not one of the aforementioned six types of barriers. The type of barrier is verbatim from the paper. | Y | Y |
| Barrier_Lat | Latitude of barrier, derived from information in paper or estimated from Google maps. | Y | Y |
| Barrier_Long | Longitude of barrier, derived from information in paper or estimated from Google maps. | Y | Y |
| Barrier_height_m | If stated in article, the average height of the barrier (measured from riverbed to crest) before the removal (unit = metres). | Y | Y |
| Barrier_width_m | If stated in article, the average width of the barrier (measured from one bank to the other) before the removal (unit = metres). | Y | Y |
| Barrier_length_m | If stated in article, the average length of the barrier before the removal (unit = metres). | Y | Y |
| Start_Removal_Year | Year removal began. | Y | Y |
| End_Removal_Year | Year removal ended. | Y | Y |
| Stage | The paper has:  both pre and post removal data (Pre/Post),  both during and post removal data (Dur/Post),  both pre and during removal data (Pre/Dur),  only pre removal data (Pre),  only post removal data (Post), or  only during removal data (Dur). | Y | Y |
| Pre_Sample _Year | Year samples were taken before barrier removal began. | Y | Y |
| Post_Sample _Year | Year samples were taken after barrier removal began. | Y | Y |
| DirectiontoBarrier | Where sample was taken in relation to the barrier (i.e. upstream or downstream). | Y | Y |
| DistancetoBarrier | Distance to the barrier if provided in the article (m). | Y | Y |
| TotalDurationofStudyYears | Number of years over which the study was completed (including first and last year of samples). | Y | Y |
| Years_since_removal | From paper or manually, number of years since removal (<1 if less than 1, 0 if during the same as removal). | Y | Y |
| InvasionStatus | Invasion status: Native (N), non-native (NN), no information (NA); this can be derived from the paper or through the *Global Register of Introduced and Invasive Species* (GRIIS) and the *Global Invasive Species Database* (GISD), if the species name and region are given. | Y | Y |
| Specieslatin_verbatim | Species name as in paper. | Y | Y |
| Speciescommon_verbatim | Common name of species as in paper. | Y | Y |
| Specieslatin_gbif | Taxonomy (GBIF). | Y | Y |
| Kingdom_gbif | Taxonomy (GBIF). | Y | Y |
| Phylum_gbif | Taxonomy (GBIF). | Y | Y |
| Class_gbif | Taxonomy (GBIF). | Y | Y |
| Order_gbif | Taxonomy (GBIF). | Y | Y |
| Family_gbif | Taxonomy (GBIF). | Y | Y |
| Genus_gbif | Taxonomy (GBIF). | Y | Y |
| Measurement | Type of measurement (e.g. species richness, abundance etc.). | N | Y |
| Unit | Unit of measurement (e.g. individuals/m2). | N | Y |
| PreSampleSize | The sample size from before the barrier removal. | N | Y |
| PrePop | The measurement of the population of the relevant species at the relevant location before the barrier removal. | N | Y |
| PrePop_SE | Standard error of population measurement before the barrier removal, if provided in paper. | N | Y |
| PrePop_SD | Standard deviation of population measurement before the barrier removal, if provided in paper. | N | Y |
| PostSampleSize | The sample size from after the barrier removal. | N | Y |
| PostPop | The measurement of the population of the relevant species at the relevant location after the barrier removal. | N | Y |
| PostPop_SE | Standard error of population measurement after the barrier removal, if provided in paper. | N | Y |
| PostPop_SD | Standard deviation of population measurement after the barrier removal, if provided in paper. | N | Y |

**Table S5.** Summary of multivariate meta-analytic models (REML; *k* = 2,840). Significant results are in bold.

| Model | Parameter | β | SE | z-value | p-value | 95% CI  (lower, upper) | Significance |
| --- | --- | --- | --- | --- | --- | --- | --- |
| Invasion Status | Native | 0.683 | 0.496 | 1.376 | 0.169 | -0.290, 1.656 | - |
|  | Non-native | 0.235 | 0.501 | 0.469 | 0.639 | -0.748, 1.217 | - |
| Invasion Status and Taxonomic Group | Native Fish | 0.469 | 0.591 | 0.792 | 0.428 | -0.691, 1.628 | - |
|  | Non-native Fish | -0.634 | 0.614 | -1.032 | 0.302 | -1.838, 0.570 | - |
|  | **Non-native Macroinvertebrates** | **3.138** | **1.483** | **2.116** | **0.034** | **0.231, 6.045** | ***** |
|  | Native Plants | 0.154 | 1.023 | 0.150 | 0.881 | -1.852, 2.159 | - |
|  | Non-native Plants | -0.043 | 1.025 | -0.042 | 0.967 | -2.052, 1.966 | - |
| Invasion Status and Direction | Native Downstream | 0.711 | 0.540 | 1.317 | 0.188 | -0.348, 1.770 | - |
|  | Non-native Downstream | 0.479 | 0.551 | 0.869 | 0.385 | -0.602, 1.560 | - |
|  | Native Upstream | 0.597 | 0.527 | 1.133 | 0.257 | -0.436, 1.629 | - |
|  | Non-native Upstream | -0.031 | 0.537 | -0.058 | 0.954 | -1.083, 1.021 | - |
| Overall years since removal (linear) | Intercept | 0.436 | 0.479 | 0.910 | 0.363 | -0.503, 1.374 | - |
|  | **Years since removal** | **0.059** | **0.023** | **2.578** | **0.010** | **0.014, 0.104** | ****** |
| Native years since removal (linear) | Intercept | 0.189 | 0.437 | 0.434 | 0.664 | -0.667, 1.045 | - |
|  | **Years since removal** | **0.068** | **0.025** | **2.742** | **0.006** | **0.012, 0.117** | ****** |
| Non-native years since removal (linear) | Intercept | 1.219 | 0.800 | 1.524 | 0.128 | -0.349, 2.787 | - |
|  | Years since removal | -0.050 | 0.045 | -1.118 | 0.264 | -0.137, 0.036 | - |
| Overall years since removal (binned) | 0-1 year | 0.444 | 0.477 | 0.930 | 0.353 | -0.492, 1.379 | - |
|  | 2-5 years | 0.654 | 0.479 | 1.366 | 0.172 | -0.285, 1.593 | - |
|  | 6+ years | 0.654 | 0.529 | 1.236 | 0.216 | -0.382, 1.691 | - |
| Native years since removal (binned) | 0-1 year | 0.191 | 0.438 | 0.437 | 0.662 | -0.667, 1.049 | - |
|  | 2-5 years | 0.425 | 0.440 | 0.967 | 0.333 | -0.437, 1.287 | - |
|  | 6+ years | 0.590 | 0.514 | 1.147 | 0.251 | -0.418, 1.598 | - |
| Non-native years since removal (binned) | 0-1 year | 1.244 | 0.795 | 1.565 | 0.118 | -0.314, 2.802 | - |
|  | 2-5 years | 1.116 | 0.799 | 1.398 | 0.163 | -0.450, 2.682 | - |
|  | 6+ years | 0.475 | 0.862 | 0.551 | 0.582 | -1.216, 2.165 | - |


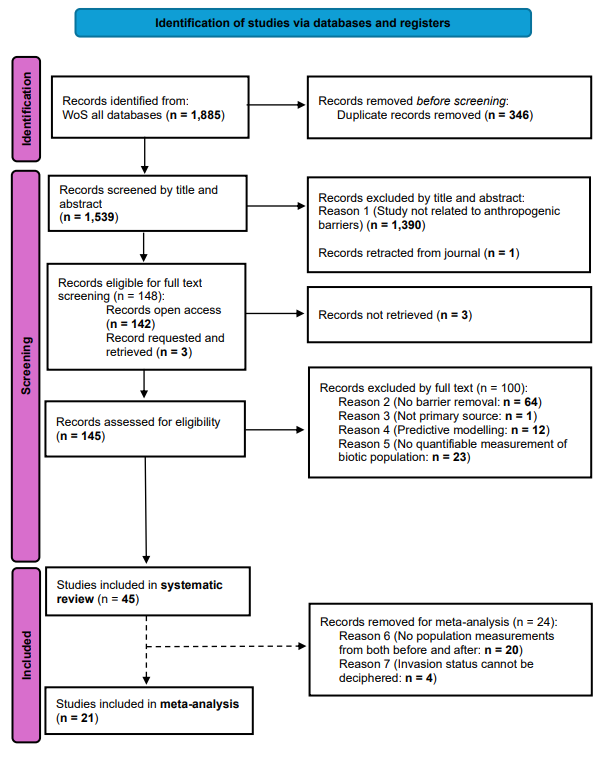


**Figure S1.** PRISMA diagram of paper inclusion and exclusion for final systematic review and meta-analysis.
